# Top-Performance Transmission Gratings with Haloalkanes-Based Polymeric Composite Materials

**DOI:** 10.3390/ma15238638

**Published:** 2022-12-03

**Authors:** Riccardo Castagna, Cristiano Riminesi, Andrea Di Donato, Oriano Francescangeli, Daniele Eugenio Lucchetta

**Affiliations:** 1 URT-CNR@UNICAM, Photonic Materials Laboratory, Università di Camerino (UNICAM), Via Sant’Agostino, 1, 62032 Camerino, Italy; 2 CNR, Institute of Heritage Science, Via Madonna del Piano, 10, 50019 Sesto Fiorentino, Italy; 3 Dipartimento di Ingegneria dell’Informazione, Università Politecnica delle Marche, Via Brecce Bianche, 60131 Ancona, Italy; 4 Dipartimento di Scienze e Ingegneria della Materia, dell’Ambiente ed Urbanistica (SIMAU), Università Politecnica delle Marche, Via Brecce Bianche, 60131 Ancona, Italy; 5 Optoacoustic Lab, Dipartimento di Scienze e Ingegneria della Materia, dell’Ambiente ed Urbanistica (SIMAU), Università Politecnica delle Marche, Via Brecce Bianche, 60131 Ancona, Italy

**Keywords:** holographic gratings, polymers, acrylate, halo-alkanes

## Abstract

We report on highly transparent holographic phase transmission volume gratings recorded in the visible region at λ = 532 nm. The maximum measured diffraction efficiency is higher than 80% with a grating pitch of Λ≈ 300 nm and a refractive index modulation Δn ≈ 0.018. To obtain these results, we used a holographic mixture based on multi-reticulated acrylate and haloalkanes (1-bromo-butane and 1-bromo-hexane) and a synergic combination of camphore-quinone, which has a maximum absorbance at c.a. 470 nm, and R6G, here used as co-initiator, to efficiently initiate the photo-polymerization process. High transparent and high efficient holographic structures based on polymers can find applications in many research fields including integrated optics, sensors, high density data storage and security.

## 1. Introduction

Many important advances in Science and Technology are obtained by using composite polymer materials [1,2,3,4,5,6,7]. Holographic gratings made by composite polymer materials are one of the most intriguing technological achievements of the last few decades [8,9,10,11,12,13,14,15]. In this context, a pivotal role is played by multi-acrylates or multi-functionalized polymer materials that, after polymerization, can give rise to highly reticulated polymer matrices. Such kinds of materials can be considered real warehouses of information brought by an incident light in terms of an interference between beams. It is worth noting as a large and ramified single monomer, that gives rise to polymer reticulation, is used for storing the information coming from an incident polymerizing light [16,17,18,19,20]. The characteristics of a grating depend on the type of application. In the sensors field, e.g., for pressure/deformation measurements [8,21,22], the following properties are usually considered: high transparency, high diffraction efficiency, high wavelength selectivity, high resolution, stable and well defined periodic structure, possibility to be functionalized making them conductive or sensitive to external chemical or biological analytes. High transparency is the signature of a grating that has low scattering losses. It is also a property related to the diffraction efficiency defined below in the text in Equation (1). The theoretical value of 100% in the diffraction efficiency means that the desired narrow range of wavelengths is totally transmitted through the sample with the same intensity as the incoming light. The direction is instead given by the Bragg angle for that specific range of wavelengths. A narrow Bragg’s peak is desired in order to best select close wavelengths. Concerning resolution, it is connected to the difficulty to write small pitches. The difficulty is usually linked from one side to the light diffraction limit and from the other side to the chemical and physical properties of the materials used. Large pitches are also a challenge in practical situations when long distances, alignment, quality of the beams are involved. Finally, concerning the sample morphology, transmission gratings can be surface relief (SR) gratings or volume gratings. SR are thin surface gratings in which the pitch is comparable with the grating thickness [19,23] while in volume gratings the thickness is much higher than the pitch linear dimensions. Concerning organic materials, reticulated acrylates are conveniently used for volume gratings, while reticulated epoxydes are usually employed for thin SR gratings. In this work, we use volume grating and acrylate-based mixtures. To initiate the photo-polymerization process in acrylate systems, free radical photo-initiators are used. In our case, in order to maintain the transparency, we opted for a combination of 2,6-Bornanedione (Camphorequinone, CQ) and (3Z)-9-[2-(Ethoxycarbonyl)phenyl]-N-ethyl-6-(ethylamino)-2,7-dimethyl-3H-xanthen-3-iminium (R6G). This is a mixture derived from similar ones used in lasing applications [24,25]. Here, we intended to promote a mutual interaction between CQ and R6G during irradiation at the level of electron transfer processes followed by free radical formation. In the present work, we decided to, furthermore, exploit the properties of di-pentaerythritol-hydroxy-penta/hexa-acrylate monomer (DPHPHA), monomer that is a multi-functional acrylate relatively large molecule. At the same time, the presence of haloalkanes in the system guarantees the transparency of the mixture, low viscosity and easy of manufacturing of the final device. In this case, however, we cannot exclude even an active participation in the photo-polymerization process of the chosen halo-alkanes [25]. Holograms showing high transparency, high values of diffraction efficiency and high angular selectivity open new perspectives for the realization of sensors for pressure and deformation measurements to be applied in many application fields such as in the reconstruction/renovation of historical/artistic buildings and/or in chemo-/bio-/medical contexts.

## 2. Materials and Methods

### 2.1. Materials

DPHPHA, 1-bromo-butane (1BB), 1-bromo-hexane (1BH) and CQ are purchased from Merck, Darmstadt, Germany; R6G from Kodak, Rochester, NY, USA.

### 2.2. Methods

In a small bottle, 69% of DPHPHA monomer, 20% of 1BH and 10% of 1BB are mixed together and stirred for ≈30 min. After stirring, 1% CQ and 0.04% R6G are added. The sample is continuously stirred for further ≈3 h at room temperature maintaining the reaction environment to be dark (periodically controlling the system) until a very low viscous pale orange mixture is obtained.

### 2.3. Sample Preparation

The so prepared syrup fills the cavity of a cell made by two microscope glass slides. The inter-glasses space of the cell is guaranteed by the use of spacers made of Mylar placed along the edges of the sandwich. Subsequently, the sample is placed in the holographic set-up for the irradiation.

### 2.4. Holographic Set-up

**Figure 1:**
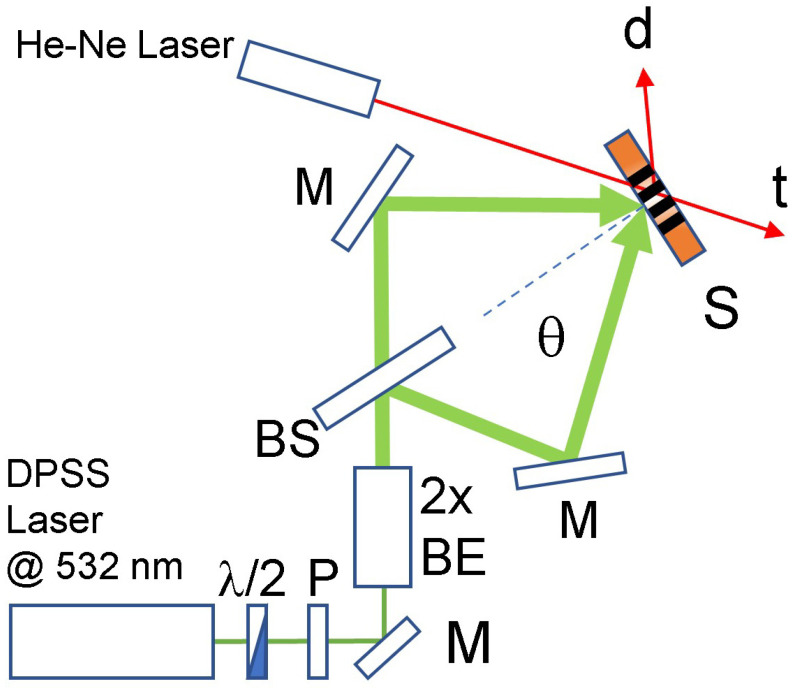
Schematic representation of the writing setup used to record the holographic one dimensional structures. λ/2 = half wavelength plate; M = mirror; P = polarizer; S = sample; 2 × BE = 2 × beam expander; t and d = transmitted and diffracted beams, respectively. The recorded periodic structure inside the sample is reported in black.

Two interfering s-polarized laser beams at λ = 532 nm are used to polymerize the samples. A sketch of the recording setup is reported in Figure 1. Each interfering beam has an intensity *I* = 150 mW/cm${}^{2}$. The interfering area has a radius *r* of ≈2.5 mm and the angle between each beam and the direction perpendicular to the glasses is θ ≈ 57°. The light intensity impinging on the sample is finely controlled by rotating a half-wave (λ/2) waveplate placed before a linear Glan Thompson polarizer (P). After that, the beam is deflected and expanded by a factor 2 using a beam expander. Finally the light is split and directed to the sample by two mirrors. An He-Ne laser impinging at the Bragg angle is used as probe to detect the grating formation. The optimal irradiation time is 1.5 s. The diffracted red spot appears immediately. During the formation of the grating, the cured parts separate from the uncured ones. The final result is a one dimensional phase grating made by polymer-rich and monomer-rich regions. From an optical point of view, this morphological arrangement corresponds to a one dimensional modulation of the refractive index of the grating itself. By illuminating the grating with an incoherent white light, the spectra of the light transmitted by the sample can be acquired. At the same time, fine angular selectivity measurements can be conducted by using a single frequency (typically a low power He-Ne laser) impinging on the sample placed on a rotating goniometer. In our experiments, we used a goniometer with a resolution of 0.036 degrees.

## 3. Results and Discussion

We used a slightly modified haloalkanes-acrylate based mixture exploited in recent works [24,25,26] to create reflection phase volume gratings for high-density optical data storage and lasing applications at $\lambda_{w}$ = 457.9 nm. In this case, in view of the development of a novel class of sensors for pressure and deformation measurements to be exploited in different contexts, we focused on the fabrication of transmission gratings polymerized at $\lambda_{w}$ = 532 nm. To initiate the photo-polymerization process, we avoided the use of highly coloured dyes/photo-initiators (red-coloured dyes, e.g., Rose Bengal) that could have resulted in high diffraction efficiency gratings but could not have led to the desired transparencies in the visible region. The absorption of the CQ is effective in the blue-region (it has a maximum absorbance at $\lambda_{w}$ = 470 nm) while the R6G absorbs in the region between 500–550 nm, with the maximum at λ = 530 nm [27]. We hypothesize a synergic effect in the photo-initiation involving, under irradiation, an electron transfer process followed by proton-transfer and a consequent production of free radicals.

The pitch of the recorded grating is selected by changing the value of the impinging angle θ. To record the grating, we used an impinging angle of ≈57°. The optimal irradiation time is 1.5 s when the light intensity is 150 mW/cm${}^{2}$ per beam. After that, the diffraction efficiency slightly increases in the dark up to its maximum value in approximately 2 s. By increasing the exposure time, we observe a reduction of the diffraction efficiency due to the appearance of a parasite grating (see [25]). The corresponding high resolution theoretical grating pitch is Λ = 318 nm. By illuminating the grating with an incoherent white light and by rotating the sample, many different colours can be selected. The samples show a very high angular selectivity, and each color can be selectivity chosen. At a glance, the colours start from near UV-A to deep Red with a very wide angular distribution. This property is desirable to obtain high resolution sensors in the near future.

**Figure 2:**
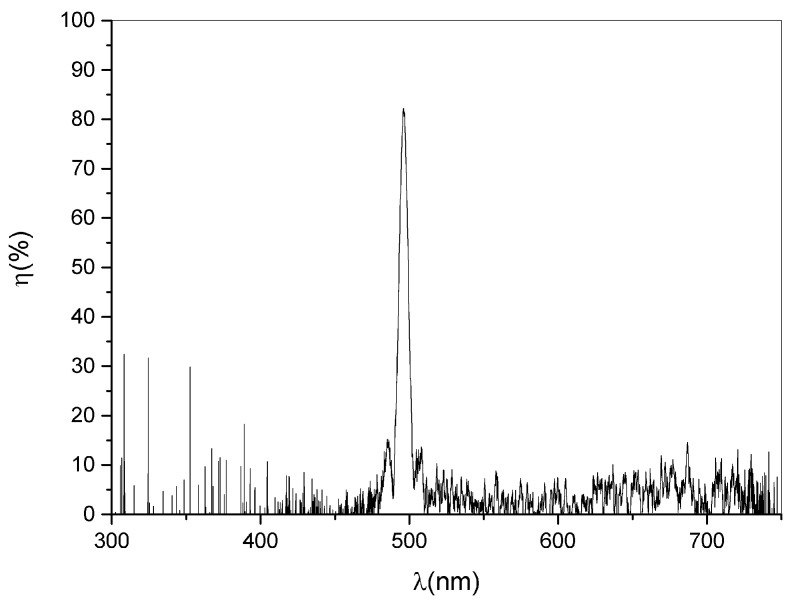
Best diffraction efficiency value measured using an incoherent white light source impinging at 56° with respect to the normal to the glasses.

**Figure 3:**
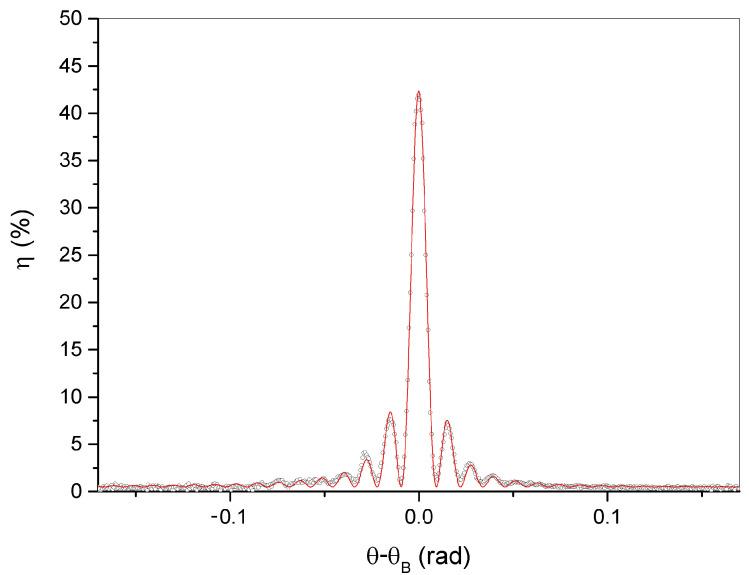
Angular selectivity measurement taken at λ = 632 nm. The experimental data fit, reported in red, is made by using Equation (1).

Each color corresponds to a Bragg angle for the diffracted wavelength. As an example, in Figure 2 a transmission spectrum showing a particularly high value of diffraction efficiency, corresponding to an incident angle of 56°, is reported. The figure shows the typical shape of the transmission peak, which is centered at ≈500 nm. The height of the peak corresponds to the diffraction efficiency value for the corresponding wavelength. The peak has a narrow FWHM of ≈8 nm. The presence of the first two lobes in the figure is remarkable. This is unusual when an incoherent and slightly divergent light beam is used to illuminate the samples. By rotating the sample holder, the grating shows a high colour selectivity. A deeper investigation is performed using a single frequency λ = 632.8 nm and the result is reported in Figure 3 for a sample showing a particularly high value of diffraction efficiency for that specific wavelenght. The FWHM is now only 0.008 rad, which is a remarkable result. The well known Kogelnik’s theory gives for the diffraction efficiency of a phase transmission grating the following expression:

(1) $$\eta \left(\nu ,\xi \right)=e^{-\frac{\alpha d}{cos\theta }}\frac{{sin\left(\sqrt{\left(\nu^{2}+\xi^{2}\right)}\right)}^{2}}{1+\frac{\xi^{2}}{\nu^{2}}}$$

in this expression, the coupling and detuning parameters are respectively defined as:

(2) $$\nu =\frac{\pi \delta nd}{\lambda cos\theta }$$

(3) $$\xi =\Delta \theta \beta dsin\theta_{0}$$

in the above equations, δn is the grating refractive index modulation, *d* the thickness of the grating, λ the free space wavelength used to read the physical properties of the recorded structures, θ the angle of incidence, $\theta_{0}$ the angle of Bragg, α the absorption coefficient, *n* the average refractive index of the mixture and β=2πn/λ. Δθ in Equation (3) is the de-phasing term which becomes effective when λ or θ are varied. The wavelength, the angle of incidence and the grating pitch Λ are linked by the following relation:

(4) $$\Delta \theta =\frac{2\pi }{\Lambda }sin\theta -{\left(\frac{2\pi }{\Lambda }\right)}^{2}\frac{\lambda }{4\pi n}$$

when the Bragg condition is fullfilled, Δθ=0, and Equation (4) can be written as:

(5) $$\theta_{0}\left(\lambda \right)=\arcsin\left(\frac{\lambda }{2\Lambda n}\right)$$

this leads to the following expression for the diffraction efficiency:

(6) $$\eta \left(\lambda ,\delta n\right)=e^{-\;\;\frac{2\alpha d}{cos\theta_{0}\left(\lambda \right)}}\;sin^{2}\left(\frac{\pi \delta nd}{\lambda cos\theta_{0}\left(\lambda \right)}\right)$$

**Figure 4:**
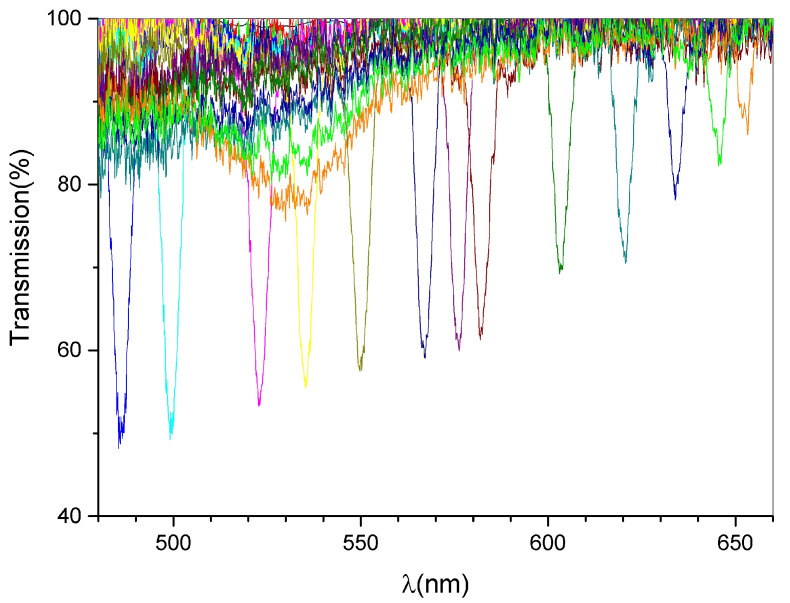
Typical transmission spectra as a function of the external rotation angles. The reflection peaks show a FWHM of ≈8 nm.

In the same figure is reported the experimental data fit made using Equation (1), which gives a refractive index modulation δn = 0.018 ± 0.004. Finally, by rotating the sample under white light illumination, we obtain different transmission spectra. A typical example is reported in Figure 4.

**Figure 5:**
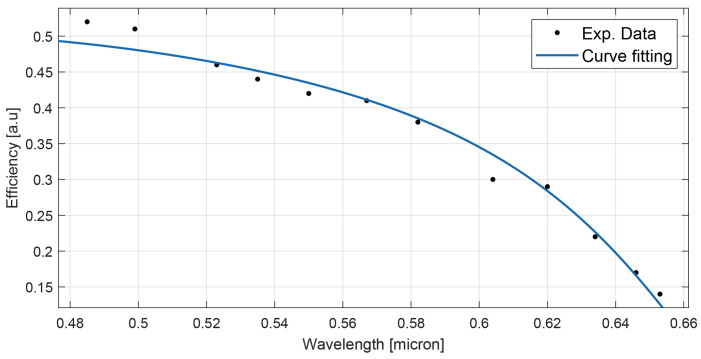
Diffraction efficiency values reported as a function of the wavelength. Each wavelength corresponds to a different Bragg angle for the diffracted signal. The continuous line represents the experimental data fit.

Each spectrum has a notch corresponding to the diffracted wavelength. The diffraction efficiency at different incident angles can be easily calculated from the figure because the height of the peak gives the value of the diffraction efficiency of the measured signal immediately [28,29]. The diffraction efficiency values derived from Figure 4 are lower than those reported in Figure 2 and Figure 3, which show the maximum of the diffraction efficiency obtained in two specific areas of the sample (typically near to the border of the spot). This is a typical behavior also observed in similar mixtures (see [30] as an example). To perform the measurements reported in Figure 4, we need to illuminate a region of the spot which responds well to the entire range of wavelengths interested during the sample rotation. This justifies the lower diffraction efficiency values reported. Figure 5 shows the values of diffraction efficiency as a function of the wavelength.

Equation (1) can be used to fit the experimental data. As we can observe, the agreement between the theory and the experiments is excellent. By assuming an average refractive index *n* = 1.48 and an effective grating thickness *d* = 10 µm, the value of the grating refractive index modulation is δn∼0.018±0.004, the absorption coefficient α=0.025±0.002 µ${}^{-1}$ and the grating pitch is Λ = 300 ± 10 nm. Results have been corrected for angle and polarization dependent Fresnel refraction.

## 4. Conclusions

In conclusion, we obtain very high efficient and narrow Bragg’s peaks related to transmission phase volume gratings. The fabrication of these one dimensional holograms potentially has many fields of application ranging from heritage science for diagnostics in reconstruction of historical and artistic buildings to active devices. Passive sensors based on holographic materials are particularly interesting when there is the necessity of a remote interrogation of the device. Active devices are useful in other fields such as in lasing applications. In any case, it is remarkable to underline the wide angular sweep of the diffracted signal connected with a high colour selectivity. This work is a further demonstration of the benefit of multi-acrylate to store the information related to the incoming polymerizing electromagnetic source.

## Data Availability

Data are available from the authors under reasonable request.
